# Special Issue “Depression: From Molecular Basis to Therapy”

**DOI:** 10.3390/ijms26083844

**Published:** 2025-04-18

**Authors:** Terezia Kiskova, Aneta Bednarova

**Affiliations:** 1 Department of Pathology, Faculty of Medicine, Pavol Jozef Safarik University and Louis Pasteur University Hospital in Kosice, 040 01 Košice, Slovakia; 2 2nd Department of Psychiatry, Faculty of Medicine, Pavol Jozef Safarik University and Louis Pasteur University Hospital in Kosice, 040 01 Košice, Slovakia

Depression, often described as the “silent epidemic” of the 21st century, stands as a complex and pervasive mental health challenge that affects millions of individuals worldwide. Major depressive disorder (MDD), a main cause of disability, is characterized by persistent sadness, hopelessness, and loss of interest in daily life [1]. Anhedonia in MDD has been associated with a worse course and outcomes and may predict the hard-to-treat manifestation [2]. This disorder affects 5% of adults [3]. The prevalence of this disorder is increasing yearly. About 300 million suffer from MDD, mainly affecting adolescents [4].

The concept of “treatment-resistant depression” (TRD) has existed since 1974 [5]; however, the past decade is characterized by a significant increase in drug developments specifically targeting patients with TRD [6].

Understanding the molecular basis of depression has become a crucial endeavor in contemporary neuroscience and psychology, as it holds the potential to unravel the mysteries behind this debilitating disorder and, ultimately, pave the way for more effective therapeutic interventions.

This Special Issue, titled “Depression: From Molecular Basis to Therapy”, contains six original papers and three reviews. They bring the newest insights into the complex topic of depression. The information included in this Special Issue forms a collection of valuable results that are supported by the newest trends and scientific facts contained in the reviews.

The treatment of patients with MDD includes the choice of standard tricyclic antidepressants, such as imipramine, amitriptyline, clomipramine, desipramine, and doxepin (they interact with neurotransmitters in the brain); selective serotonin reuptake inhibitors (SSRIs), including fluoxetine, sertraline, paroxetine, and escitalopram; and serotonin/norepinephrine reuptake inhibitors (SNRIs), including milnacipran, DXT, DVS, or venlafaxine [1]. However, about one-third of patients with depression do not adequately respond to antidepressant pharmacotherapy [7]. That is why it is a necessity to develop new strategies or drugs to treat depression more effectively.

Vagus nerve stimulation (Figure 1) may represent a long-term adjunctive treatment option in patients with TRD, as referred to in Ref. [8]. The authors collected data from the peripheral blood of six patients. They have identified cytokine changes from baseline to six months and long-term responses (12 months) with significant correlations [8].

Another novelty approach may utilize the pyridoindole derivative called SMe1EC2M3. The authors tested its effects during stress-induced depression in Wistar–Kyoto male rats as an animal model of TRD. They found out that during behavioral testing, such as the open-field test, novel object recognition test, or forced swim test, SMe1EC2M3 ameliorated behaviors associated with TRD [9]. Gyrophoric acid, a lichen secondary metabolite, was used for the first time ever during immobility–restriction-induced depression in male Wistar rats. The authors showed its antidepressant effect, together with the stimulation of hippocampal neurogenesis concomitantly with the increased number of mature neurons, indicating the role of gyrophoric acid in the maturation process of neurons [10]. Chronic stress, used in both preclinical studies, has been found to be the major risk factor in the development of depression. Rodent models are commonly used to explore the molecular factors and triggers of chronic stress and drugs in the central nervous system. As referred to in previous experimental studies, stress results in physical and behavioral changes in individuals, including weight or temperature changes [11].

An important aspect that should be discussed is the fact that antidepressant therapy is associated with weight changes. This is very often the reason for treatment withdrawal. This aspect is probably caused by patho-mechanisms such as the abnormal functioning of the homeostatic (mostly humoral) and hedonic (mostly dopaminergic) circuits of appetite regulation, as well as causing neuromorphological and neurophysiological changes underlying the development of depression [12].

The newly discussed topic is the gut–brain axis, which affects a lot of (if not all) diseases. Moreover, special attention is focused on the therapeutic strategies through the microbiota–gut–brain axis. It is known that symbiotic interactions between the gut microbes and the host can affect mental health [13]. One interesting way to ameliorate neuropsychiatric behaviors induced by chronic sleep deprivation is an exogenous melatonin administration. Li et al. (2024) revealed that sleep deprivation in rats led to anxiety and depression accompanied by cognitive decline. They found out that melatonin may influence brain messaging through the microbiota–gut–brain axis by increasing the production of short-chain fatty acids and decreasing the production of secondary bile acids [14].

It is well-known that seasonal effects have a significant impact on the pattern of recurrence or hospitalization of bipolar disorders. Dallaspezia et al. (2024) retrospectively studied neutrophile-to-lymphocyte ratios (NLRs) in 824 MDD participants; 255 patients were affected by obsessive–compulsive disorders and 248 were healthy individuals. It has been shown that autumn and winter are the seasons that influence bipolar disorder-associated systemic inflammation when compared to other patients and healthy individuals. The authors suggest that seasonal rhythms should be considered when managing antidepressant treatments (especially immunomodulatory treatments) as a part of the precision medicine approach [15].

Genetic and epigenetic predispositions that can unexpectedly manifest at any point in the life of a patient suffering from depression also pose an issue. The *KALRN* gene (which encodes kalirin) has been shown to play a role in several neuropsychiatric and neurodegenerative disorders. Thus, the authors tested the association between depressive symptoms in patients and epigenetic variability in *KALRN*. They revealed that there is no association between methylation levels and cognitive performance of depressive symptoms. However, they revealed that there is an association between interindividual variations in depressive symptoms and a region within the *KALRN* promoter, namely cg13549966 (CHRZ3:124106738). Thus, epigenetic variations may play a significant role in the manifestation of atypical depression [16].

## Conclusions

Many research groups are searching for biomarkers and appropriate therapeutic approaches for the treatment of MDD. Predicting the most effective antidepressant treatment remains a challenging process that is complicated due to the potential risk of serious adverse reactions. Individuals with suicidal behavior also remain at risk. The above-mentioned research studies open further discussions crucial for the future understanding of MDD.

## Figures and Tables

**Figure 1:**
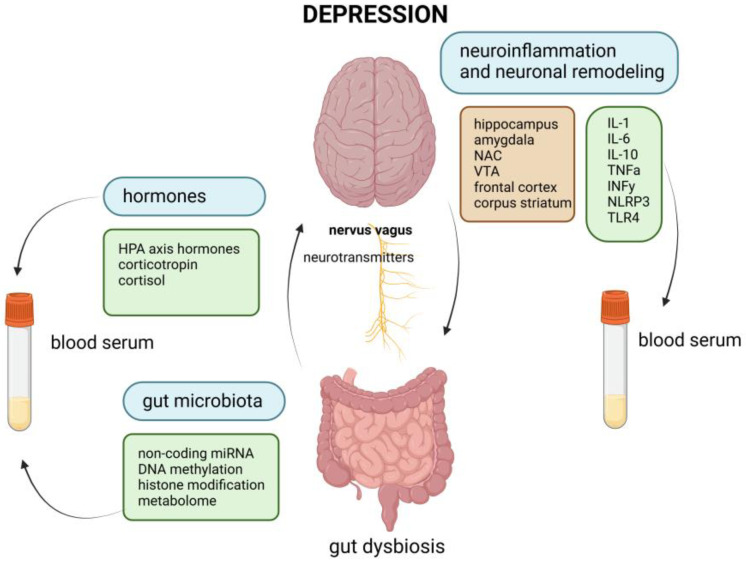
The various mechanisms involved in the pathophysiology of depression. VTA—ventral tegmental area; NAC—nucleus accumbens; MSNs—medium spiny neurons; TNFα—tumor necrosis factor alpha; IL—interleukin; TLR—Toll-like receptor; INFγ—interferon gamma; NLRP—nucleotide-binding oligomerization domain, leucine-rich repeat, and pyrin-containing domain. Created with BioRender.com.

## Abbreviations

The following abbreviations are used in this manuscript:

MDPI	Multidisciplinary Digital Publishing Institute
DOAJ	Directory of open-access journals
TLA	Three-letter acronym
LD	Linear dichroism
